# Postnatal Light Effects on Pup Stress Axis Development Are Independent of Maternal Behavior

**DOI:** 10.3389/fnins.2017.00046

**Published:** 2017-02-10

**Authors:** Georgia Coleman, Maria M. Canal

**Affiliations:** Division of Neuroscience and Experimental Psychology, School of Biological Sciences, Faculty of Biology, Medicine and Health, University of Manchester, Manchester Academic Health Science CentreManchester, UK

**Keywords:** corticosterone, Arginine-vasopressin, Corticotrophin-releasing hormone, glucocorticoid receptor, constant light, constant darkness, licking and grooming

## Abstract

Postnatal environment shapes brain development during key critical periods. We have recently found that postnatal light environment has long-term effects on the stress and circadian systems, which can lead to altered stress responses, circadian behavior and a depressive phenotype in adulthood. However, it is still unclear how light experience affects the postnatal development of specific stress markers in the pup brain and the role played by maternal behavior and stress. To test this, we raised mice under either light-dark cycles (LD), constant light (LL) or constant darkness (DD) during the suckling stage. After weaning, all mice were exposed to LD until adulthood. Results show that postnatal light environment does not have any significant effects on dam stress levels (plasma corticosterone concentration, Arginine-vasopressin and Glucocorticoid receptor (GR) protein expression in the brain) or maternal behavior, including licking and grooming. Light environment does not have a major effect on litter characteristics or pup growth either. Interestingly, light environment during the suckling stage significantly impacted Corticotrophin-releasing hormone (CRH) and *Gr* mRNA expression in pup brain during development. Furthermore, a difference in *Crh* mRNA expression between LL- and DD-raised mice was still observed in adulthood, long after the exposure to abnormal light environments had stopped. Taken together, these data suggest that the long-term effects of postnatal light environment on the pups' stress system cannot be attributed to alterations in either maternal behavior and/or stress axis function. Instead, postnatal light experience may act directly on the pup stress axis and/or indirectly via circadian system alterations.

## Introduction

The importance of early life programming on later health and wellbeing is becoming increasingly evident, particularly in the fields of stress and obesity. An important aspect of early programming is the long-term effects of postnatal light experience on brain development. We and others have demonstrated that postnatal light environment impacts adult circadian behavior and responses to light and that these effects reflect long-term changes in circadian clock gene and neuropeptide expression at the level of the suprachiasmatic nucleus (SCN) of the brain hypothalamus, site of the principal circadian pacemaker in mammals (Rusak and Zucker, 1979; Smith and Canal, 2009; Ciarleglio et al., 2011a; Brooks et al., 2014). Furthermore, recent evidence suggests that, in addition to the effects on the circadian system, postnatal light experience will also induce long-term changes on the stress system, the hypothalamic-pituitary-adrenal (HPA) axis, and mood regulation. Specifically, compared to mice raised under control 24-h light-dark (LD) cycles, mice raised under constant light (LL) or constant darkness (DD) during the first 3 postnatal weeks will show decreased glucocorticoid receptor (GR) expression in the hippocampus, increased plasma corticosterone concentration at the onset of the dark phase of a LD cycle and a depressive phenotype in adulthood (Coleman et al., 2016). In line with this, Ciarleglio et al. found that mice raised under long (summer-like) photoperiods displayed shorter free-running locomotor activity rhythms, together with increased anxiety-like behaviors compared to mice raised under short (winter-like) photoperiods, which were more prone to depressive-like behaviors in adulthood (Ciarleglio et al., 2011a,b). However, the mechanisms underlying postnatal light experience effects on the developing circadian and stress systems are still unclear.

Maternal rhythm is not essential for the development of normal pup rhythm (Reppert and Schwartz, 1986; Jud and Albrecht, 2006), or for the long-term effects of LL-rearing on the pups' locomotor activity rhythms (Cambras et al., 1997). However, arrhythmic dams have altered maternal care which can result in attenuated pup growth and decreased pup survival (Hoshino et al., 2006). Furthermore, numerous studies have highlighted the importance of maternal care in the development of offspring stress response, both in rodents and humans (Korosi and Baram, 2009). Disrupted maternal care can lead to increased neuroendocrine responses to stress as well as an increased predisposition to mental health illnesses (Heim et al., 2008). One important aspect of maternal care affecting the development of the HPA axis in rodent offspring is licking and grooming behavior: decreased maternal licking and grooming leads to an increased stress profile in the offspring later in life (Francis and Meaney, 1999; Meaney, 2001). Furthermore, LL exposure can be seen as a stressful environment for rodents (Claustrat et al., 2008) and maternal stress has been shown to also alter maternal care (Ivy et al., 2008). Therefore, it is possible that exposure to an altered light environment such as LL or DD during the suckling period can affect maternal circadian and caring behaviors, which can then impact the development of pup HPA axis and future stress regulation.

The main aim of this study was to determine the effects of the light environment in which litters are kept during the suckling stage on maternal behavior and stress levels, together with pup growth and stress axis development. For this, we exposed dams and their litters to either 24-h LD cycles, DD or LL from birth until weaning, when all pups were then kept in LD. We examined maternal and pup behavior throughout the suckling stage; maternal plasma corticosterone concentration and Arginine-vasopressin (AVP) and GR expression in the brain on the day of weaning; litter characteristics, pup growth and pup *Corticotrophin-releasing hormone, Avp*, and *Gr* mRNA expression development in the brain, from birth until adulthood.

## Materials and methods

### Animals

Mice were maintained under a controlled ambient temperature of 21 ± 1°C, with water and food (B&K Universal, Hull, UK) available *ad libitum*. Mean light intensity was 330 μW/cm^2^ at cage floor level provided by white LED light, while darkness consisted of no light (0 μW/cm^2^), unless stated otherwise. Cages were changed every fortnight at random times during the day. Infrared goggles were used in the routine maintenance of animals in the dark.

All experiments followed the same protocol: pregnant C57BL/6J mice raised in our colony were transferred to either 12:12 h light:dark cycles (LD), constant light (LL) or constant dark (DD) environments 2-3 days before their predicted delivery date (postnatal day 0, P0), to ensure that all pups were born under the intended light conditions. From P0 until P21, suckling stage, the dam and her litter remained in the same light condition. At P21, offspring of all experimental groups were weaned; siblings were separated by sex and group-housed under 24-h LD cycles until the end of the experiment. During the suckling stage, the dam's locomotor activity was continually recorded (see below).

Throughout the experiments, adequate measures were taken to minimize pain or discomfort of the animals. All experimental procedures were conducted in accordance with the United Kingdom Animals (Scientific Procedures) Act 1986 following approval by the local Ethics Committee.

### Locomotor activity recording

Locomotor activity was recorded continuously, from P0 until P21, using infrared activity meters placed outside of the mouse cage, as described previously (Smith and Canal, 2009). Data were collected in 15 min bins, stored on a computer and analyzed using El Temps© (A Diez-Noguera, University of Barcelona, Barcelona, Spain). In animals kept under LL or DD environments, the onset of the locomotor activity rhythm (circadian time 12, CT12) was used as a reference point for further calculations. The characteristics of the dams' locomotor activity rhythms were analyzed: The period was calculated using the X^2^-periodogram (Sokolove and Bushell, 1978). The percentage of variance explained by the highest peak in the periodogram (PV value) was used as an indicator of the stability/strength of the circadian rhythm of locomotor activity.

### Exp 1

The aim of the first experiment was to determine the effect of abnormal light environments during the suckling stage on the dam. We examined evolution of dam body weight and mother-pup behavior during the suckling period. In addition, we examined various stress markers (plasma corticosterone concentration, AVP and GR expression in the brain) on the dam, on the day of pup weaning.

A total of 14 pregnant C57BL/6J mice raised in our colony were transferred to either LD, LL, or DD environments 2–3 days before their predicted delivery date (P0). Dam body weight and mother-pup behavior were examined at P6, P10, and P14 (see below). At weaning (P21), dams were culled in their respective lactation light environments under isoflurane anesthesia. The culling times were Zeitgeber Time 5 (ZT5, corresponding to 7 h before lights off) for dams kept under LD cycles and CT5 (corresponding to 7 h before onset of activity), for dams kept under either LL or DD environments. Trunk blood and brains were rapidly collected fur further processing (see below).

#### Mother-pup behavior

Mother-pup behavior was measured at three ages in each litter throughout the suckling stage; P6, P10, and P14 with the same protocol followed at each age. Behavior was observed for two lots of 15 min bouts, one starting at ZT/CT4 (test 1) and the next, starting an hour after the first recording finished (ZT/CT5.25, test 2). These times were chosen as most nursing behavior usually occurs during the light/inactive period in mice (Shoji and Kato, 2006). On the day prior to a recording day, locomotor activity was used to predict CT4 for LL and DD groups. Behavior in the dark was recorded under dim red light (54 μW/cm^2^). At the start of test 1, litters and mothers were weighed and a pup retrieval test was carried out. The pup retrieval test involved placing the litter at the other end of the cage to the nest and measuring the time it took the mother to retrieve all of her pups back to the nest. Then maternal and pup behaviors were scored following a protocol adapted from Lyst et al. (2012). The following maternal behaviors were scored every 20 s throughout the 15 min recording period: nest building, nursing, suckling, resting on the nest, feeding and drinking, resting outside the nest and other active. In addition, a selection of behaviors was also recorded whenever they happened throughout the 15 min observation period: maternal behaviors (pup retrieve, licking and grooming, autogroom) and pup behaviors (suck attempt, play fight, autogroom, allogroom). An hour after the end of test 1, test 2 would commence, which involved scoring of maternal and pup behaviors for 15 min (similar to test 1), but did not include an initial pup retrieval test.

#### Corticosterone assay

Blood was processed for plasma corticosterone analysis (Cayman chemicals, Michigan, USA) following the manufacturer's protocol as previously described (Coleman et al., 2016).

#### Immunohistochemistry

Immunohistochemistry was performed as previously described (Smith and Canal, 2009). Anti-rabbit AVP was used at a concentration of 1:10 000 (Fitzgerald Industries International, Inc., Concord, MA, USA) and anti-rabbit GR (Santa Cruz Biotechnology, Dallas, TX, USA) at a concentration of 1:5000. For both experiments, biotinylated IgG goat anti-rabbit was used as the secondary antibody, at a concentration of 1:400 (Vector laboratories, Inc., Burlingame, CA, USA). Control sections were also processed without addition of either the primary or secondary antibody to test specificity and these showed no staining. Sections were visualized using a microscope (Leica DM2000, Leica Microsystems, Milton Keynes, UK). Digital images were captured, displayed on a computer screen and both the number of immune-positive cells and the optical density were manually counted using Image J (v. 1.48, NIH, Bethesda, US). Two researchers blind to the experimental group selected the region of interest as either paraventricular nucleus of the hypothalamus (PVN), *Cornu Ammonis* 1 (CA1) or Dentate Gyrus (DG) regions of the hippocampal formation according to the mouse brain atlas (0.22–3.16 mm posterior to bregma (Paxinos and Franklin, 2004) and counted the number of immune-positive cells in each region of interest (left and right brain, *n* = 4–6 sections per animal). Their average was used for further analysis. An investigator naïve regarding experimental group determined the optical density of AVP and GR staining by drawing a line around PVN, CA1 and DG boundaries and subtracting the background level of staining. An average of the levels of optical density for each section and each mouse was calculated. The group average was then obtained.

### Exp 2

The aim of the second experiment was to determine the effects of abnormal postnatal light environments on the pups' growth and development of stress markers in the brain. We examined evolution of pup body weight, average number of pups per litter, percentage of males per litter and development of *Avp, Crh*, and *Gr* mRNA expression in the PVN.

A total of 59 pregnant C57BL/6J mice raised in our colony were transferred to either LD, LL or DD environments 2–3 days before their predicted delivery date (P0). The dam's locomotor activity was used to calculate the time of pup culling for further qPCR analysis (see below). During the suckling stage, mice were culled at P1, P10, and P20. At weaning (P21), siblings were separated by sex, group-housed and kept under LD until the end of the experiment (P50). Culling times were CT/ZT 4 and CT/ZT16 at each age. Culls in the dark were carried out under dim red light (8.7 μW/cm^2^).

#### Quantitative polymerase chain reaction (qPCR)

Only male mouse brains were used for qPCR analysis. qPCR was performed as previously described (Coleman et al., 2016). Briefly, flash-frozen 250 μm thick sections were cut in a cryostat and mounted on slides. For each mouse, 3 slides containing the PVN were used. The PVN was located using a brain atlas (0.58–1.06 mm posterior to bregma (Paxinos and Franklin, 2004). Punches containing the PVN were collected into RNAse free tubes then stored at −80°C until extraction. RNA was extracted using RNeasy micro kits (Qiagen, Manchester, UK) following the manufacturer's protocol. Extracted RNA samples were tested on the nanodrop (Thermo Scientific, Warrington, UK) and only samples that had a 260/280 ratio higher than 1.7 were used, which led to three samples being discounted from further analysis. cDNA was obtained by reverse transcription (RT) reaction of the extracted RNA samples using the High Capacity RNA-to-cDNA kit available from Applied Biosystems (Life Technologies, Paisley, UK) resulting in a 20 μL reaction volume. Non RT controls were run alongside experimental samples. Samples were then stored at −20°C until used.

qPCR experiments were run in triplicate using SYBR Green (Power SYBR Green PCR master mix, Applied Biosystems, Life Technologies, Paisley, UK) on a 7900HT Fast Real-Time PCR system machine (Applied Biosystems, Life Technologies, Paisley, UK). The nucleotide sequences (5′–3′) of the primers used were: *Avp*, TCTGACATGGAGCTGAGACAG (F) and GGCAGGTAGTTCTCCTCCTG (R); *Crh*, CTCTCTGGATCTCACCTTCCA (F) and ATCTCCATCAGTTTCCTGTTGCT (R); *Gr*, TTCTGTTCATGGCGTGAGTACC (F) and CCCTTGGCACCTATTCCAGTT (R). Melt curve analysis was performed to check that only one product was being amplified and the qPCR product was run on an agarose gel to confirm specificity by showing one band at the expected size. *Gapdh* was quantified as endogenous control. The nucleotide sequences (5′–3′) of the *Gapdh* primers used were: TGTGTCCGTCGTGGATCTGA (F) and CCTGCTTCACCACCTTCTTGA (R). In addition, *Gapdh* was also used as the normalizing reference gene: *Avp, Crh*, and *Gr* gene expression were normalized to that of *Gapdh* in each sample. Relative levels of the genes of interest were determined using the 2-ΔΔCt method (Schmittgen and Livak, 2008). Data are expressed as percentage relative to the LD-raised, P1, ZT4 group.

### Statistical analysis

Data were analyzed by means of an ANOVA of general linear models using SPSS Statistics (v. 22, IBM, Armonk, NY, USA). The independent variables were: suckling stage light environment (LD, LL or DD), age/postnatal day, CT/ZT (if applicable), sex (if applicable). The dependent variables were: period and PV of the dam's locomotor activity rhythm, duration of pup retrieval test, dam's body weight, plasma corticosterone concentration, AVP optical density in the PVN, number of AVP+ cells per mm^2^ in the PVN, GR optical density in the PVN, number of GR+ cells per mm^2^ in the PVN, GR optical density in the CA1, number of GR+ cells per mm^2^ in the CA1, GR optical density in the DG, number of GR+ cells per mm^2^ in the DG, number of pups per litter, percentage of male pups per litter, pup body weight, *Avp* expression in the PVN, *Crh* expression in the PVN and *Gr* expression in the PVN. For all models, interactions between the independent variables were also tested. When a statistically significant difference was encountered (*p* < 0.05), a Bonferroni post hoc test was applied.

We analyzed the behavioral scoring data with a Kruskal-Wallis non-parametric test, using suckling stage light environment, age and test number as independent variables. The dependent variables were all the behaviors scored (see above). When a significant effect was found, a Mann-Whitney U test was run to compare individual groups. A sequential Bonferroni–type correction for multiple comparisons was used (Benjamini and Hochberg, 1995). Results are expressed as average ± standard deviation unless otherwise stated.

## Results

### Exp 1—suckling light environment effects on the dam

#### Dam body weight and stress markers

**Figure 1:**
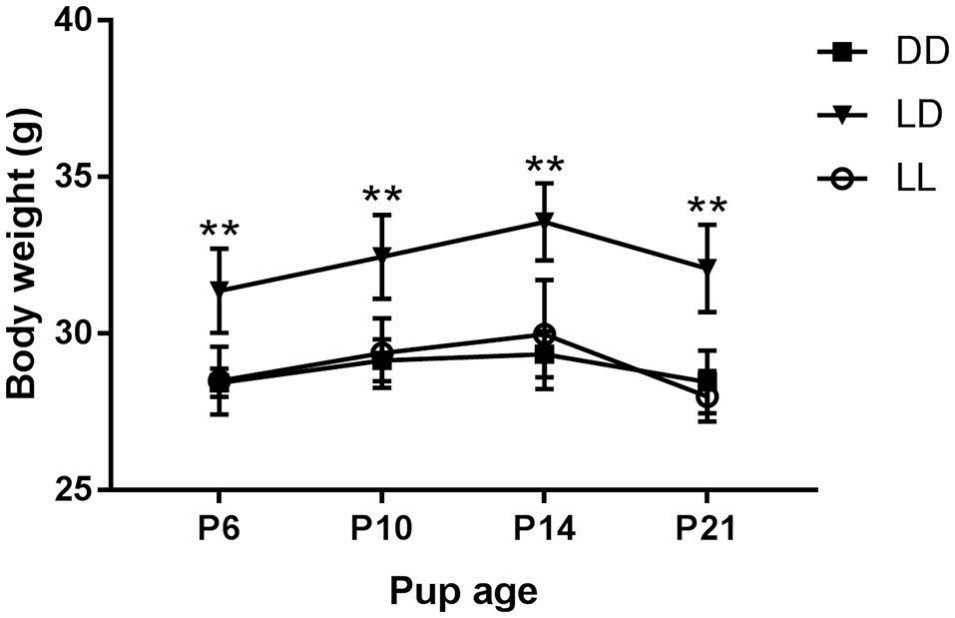
**Dam body weight during the suckling stage**. Body weight of dams kept either under constant darkness (DD), 24-h light-dark cycles (LD) or constant light (LL) during the suckling stage. P, postnatal day. ^**^p < 0.005 LD vs. DD, LL. Data are presented as average ± SEM (*n* = 3–6).

We found that the dams' body weight was dependent on light environment [*F*_(2, 11)_ = 10.359, *p* < 0.001], with dams kept under LD having higher body weights than those kept under DD or LL (*p* < 0.005 for both comparisons, Figure 1). We found no effect of pups' postnatal age on dams' body weight (*p* > 0.05).

The dams' plasma corticosterone concentration on the day of pup weaning was independent on the light environment they had been exposed to during the suckling stage (LD: 40.06 ± 4.38 ng/mL; DD: 68.85 ± 40.02 ng/mL; LL: 32.31 ± 5.87 ng/mL). Similarly, light environment during the suckling stage had no effect on AVP and GR protein expression in the PVN, CA1, and DG regions of the dam brains (Table 1).

**Table 1:** **Dam stress markers expression**.

			**DD**	**LD**	**LL**
PVN	AVP	*N* cells/100,000 μm^3^	1.38 ± 0.10	1.50 ± 0.11	1.48 ± 0.10
		OD (a.u.)	25.45 ± 5.97	29.24 ± 3.35	29.60 ± 3.79
	GR	*N* cells/100,000 μm^3^	8.47 ± 0.76	8.90 ± 1.22	9.70 ± 0.70
		OD (a.u.)	9.64 ± 3.59	9.88 ± 2.76	9.39 ± 1.73
CA1	GR	*N* cells/100,000 μm^3^	13.77 ± 2.23	17.48 ± 3.31	17.97 ± 4.34
		OD (a.u.)	23.65 ± 3.26	18.99 ± 3.12	19.21 ± 1.08
DG	GR	*N* cells/100,000 μm^3^	20.90 ± 1.93	26.02 ± 4.11	25.68 ± 3.62
		OD (a.u.)	24.64 ± 8.43	30.54 ± 11.25	28.81 ± 6.62

*Protein expression in the brain of dams kept either under constant darkness (DD), 24-h light-dark cycles (LD), or constant light (LL) during the suckling stage. AVP, arginine vasopressin; CA1, Cornu Ammonis 1; DG, dentate gyrus; GR, glucocorticoid receptor; OD, optical density; PVN, paraventricular nucleus of the hypothalamus. Data are presented as average ± SD (n = 3–6)*.

#### Dam behavior

**Figure 2:**
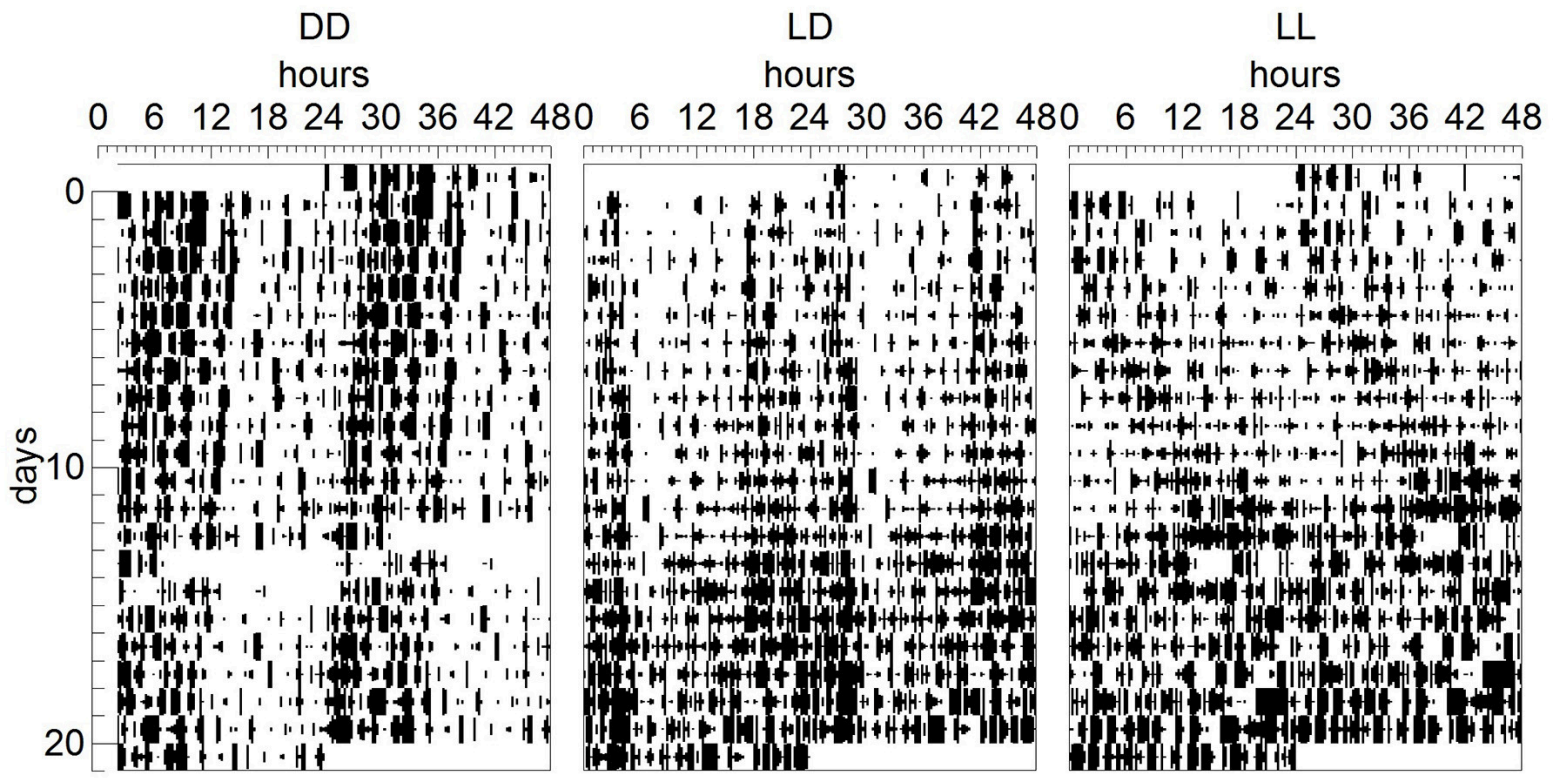
**Dam locomotor activity rhythm during the suckling stage**. Representative double-plotted actograms showing the daily motor activity pattern of dams kept either under constant darkness (DD), 24-h light-dark cycles (LD) or constant light (LL) during the suckling stage. In ordinates, experimental days are represented.

The dams' locomotor activity was recorded from pup birth until weaning. As expected (Cambras et al., 1997), the locomotor activity rhythm became slightly more difficult to detect toward the end of the suckling period, when pups grew and started moving around the cage, masking the dam's rhythm. Dams exposed to LL showed weak circadian rhythms of locomotor activity which did not reach statistical significance, but were visible enough to calculate CT12 in the mother-pup behavior experiments at P6, P10, and P14 (Figure 2). All dams exposed to LD and DD showed significant circadian rhythms of locomotor activity, with the free-running rhythm of the dams in DD being significantly shorter (23.88 ± 0.05 h) than that of dams in LD [24.0 ± 0 h, *F*_(1, 7)_ = 35.000, *p* = 0.01, Figure 2]. There were significant differences in PV according to light environment [*F*_(2, 10)_ = 7.939, *p* = 0.009]. *Post-hoc* test showed that dams exposed to LL had significantly lower PV values than those exposed to LD (*p* = 0.009): LD: 10.52 ± 1.23%; DD: 8.97 ± 4.05%; LL: 4.34 ± 0.42%.

In the pup retrieval test, we found that retrieval duration was similar in all groups and thus independent on both the age of the pups (P6, P10) and the light environment in which the litters were raised (LD: 2.4 ± 0.9 min; DD: 3.3 ± 2.2 min; LL: 2.4 ± 0.7 min). We were unable to measure pup retrieval at P14 because at this age pups moved freely and quickly around the cage.

**Figure 3:**
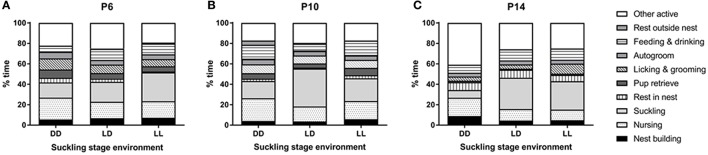
**Dam behavior during the suckling stage**. Dams' behavior was observed at 3 time-points during the suckling stage; at pups' postnatal day (P) 6 **(A)**, P10 **(B)**, and P14 **(C)**. The dams and their litters were kept either under constant darkness (DD), 24-h light-dark cycles (LD) or constant light (LL). Data are presented as average (*n* = 3–6).

With regards to dam behavior, nest building, suckling and autogroom behaviors were independent on light environment during the suckling stage, age of the pups and test number. Only other active behavior was found to depend on light environment (*p* < 0.05), with DD-exposed dams showing more other active behavior than LL-exposed dams (*p* < 0.01, Figure 3). Resting in the nest was dependent on age (*p* < 0.01), with dams resting in the nest more at P14 than at P6 and P10 (*p* < 0.001 for both comparisons, Figure 3). Resting outside the nest also depended on age (*p* < 0.05), with dams resting more outside the nest at P10 than at P14 (*p* < 0.01, Figure 3). The number of pup retrievals was also dependent on age (*p* < 0.05), with more retrievals happening at P6 than at P14 (*p* < 0.01, Figure 3). With regards to test number, we found that nursing, pup retrieval and licking and grooming behaviors were augmented in the first test compared to the second test (*p* < 0.001 for all comparisons), while feeding and drinking were increased during the second test compared to the first test (*p* < 0.001). In addition, we found significant interactions between pup age and test number for nursing, pup retrieval and licking and grooming behaviors (*p* < 0.01 for all comparisons). Specifically, we found that only in the first test, an effect of age was seen: nursing behavior and pup retrieval were decreased at P14 compared to P6 and P10 (*p* < 0.005 for all comparisons) and licking and grooming was decreased at P14 compared to P6 (*p* < 0.01, Figure 3).

### Exp2—suckling light environment effects on the pup

#### Effects on litter characteristics and pup body weight

The number of pups per litter was found to be independent on postnatal light environment (LD: 6.1 ± 1.8 pups/litter; DD: 5.8 ± 1.4 pups/litter; LL: 5.7 ± 2.0 pups/litter). The percentage of males per litter was also independent on postnatal light environment (LD: 53.5 ± 26.5%; DD: 56.2 ± 19.7%; LL: 47.7 ± 18.8%).

**Figure 4:**
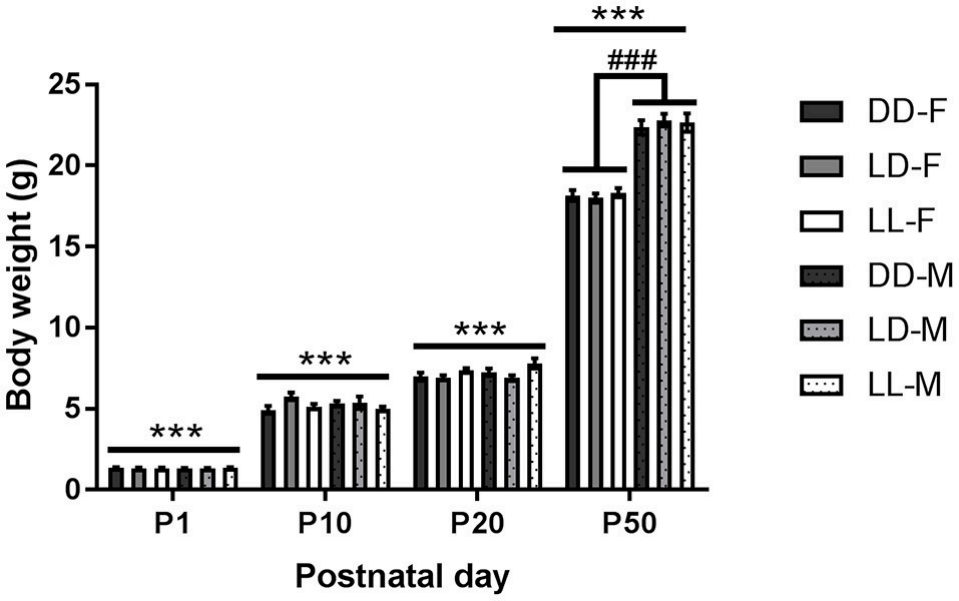
**Pup body weight throughout the experiment**. Body weight of pups kept either under constant darkness (DD), 24-h light-dark cycles (LD) or constant light (LL) during the suckling stage (postnatal day, P0-P21) and kept in LD thereafter. F, females; M, males. ^***^p < 0.001 between ages, ^###^*p* < 0.001 F vs. M. Data are presented as average ± SEM (*n* = 6–23).

Pup body weight increased significantly with age [*F*_(3, 23)_ = 5378.780, *p* < 0.001], with all ages different from each other (*p* < 0.001, Figure 4). We found a significant interaction between age and sex [*F*_(3, 23)_ = 110.441, *p* < 0.001], so that only at P50, there was a significant sex difference, with males weighing more than females (*p* < 0.001, Figure 4). In addition, we found a significant interaction between age and lactation [*F*_(6, 23)_ = 2.757, *p* < 0.05]: at P10, LD-raised mice weighed more than DD- and LL-raised mice (*p* < 0.01 for both comparisons); while at P20, LD-raised mice weighed less than LL-raised mice (*p* < 0.01).

#### Pup behavior

**Figure 5:**
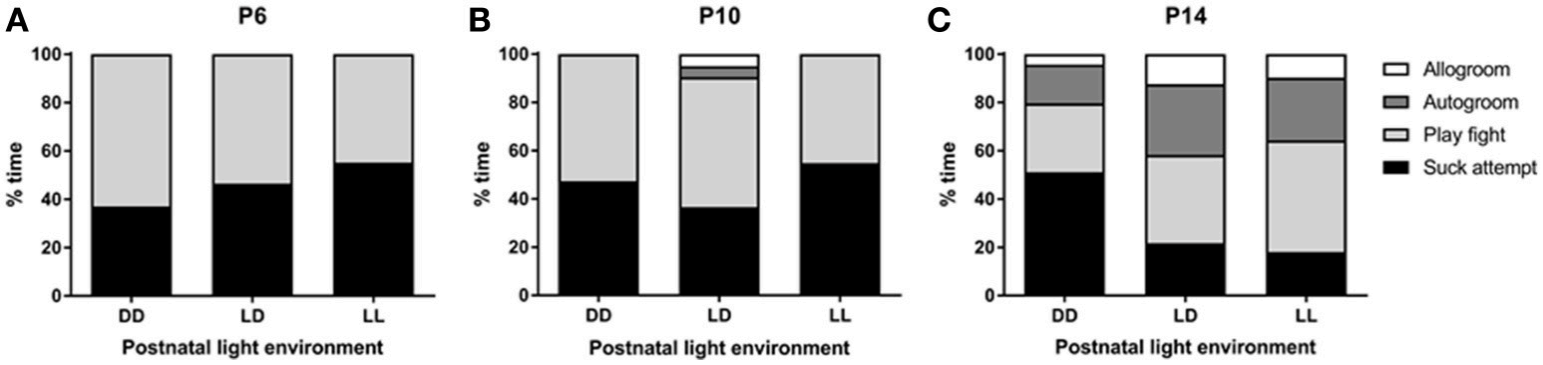
**Pup behavior during the suckling stage**. Pups' behavior was observed at 3 time-points during the suckling stage; at postnatal day (P) 6 **(A)**, P10 **(B)**, and P14 **(C)**. The litters were kept either under constant darkness (DD), 24-h light-dark cycles (LD) or constant light (LL) throughout the suckling stage. Data are presented as average (*n* = 6–12).

We found no significant effect of light environment, postnatal age or test number in the number of suck attempts recorded (Figure 5). Allogroom depended on the age of the animal, so that the number of times that pups groomed each other was higher at P14 compared to P6 and P10 (*p* < 0.001 for both comparisons). An interaction between age and lactation was found for autogroom, in that only in LD- and LL-raised mice, the number of times that pups groomed themselves was higher at P14 compared to P6 and P10 (*p* < 0.005 for both comparisons). Postnatal light environment had a significant effect on the amount of play-fight between pups (*p* < 0.05), with LL-raised mice showing more play-fight than DD-raised mice (*p* < 0.01, Figure 5). Test number was only found to be significant with regards to play-fight, so that the amount of play-fight was higher when the mice were observed in the second test compared to the first test (*p* < 0.05).

#### Pup stress markers

**Figure 6:**
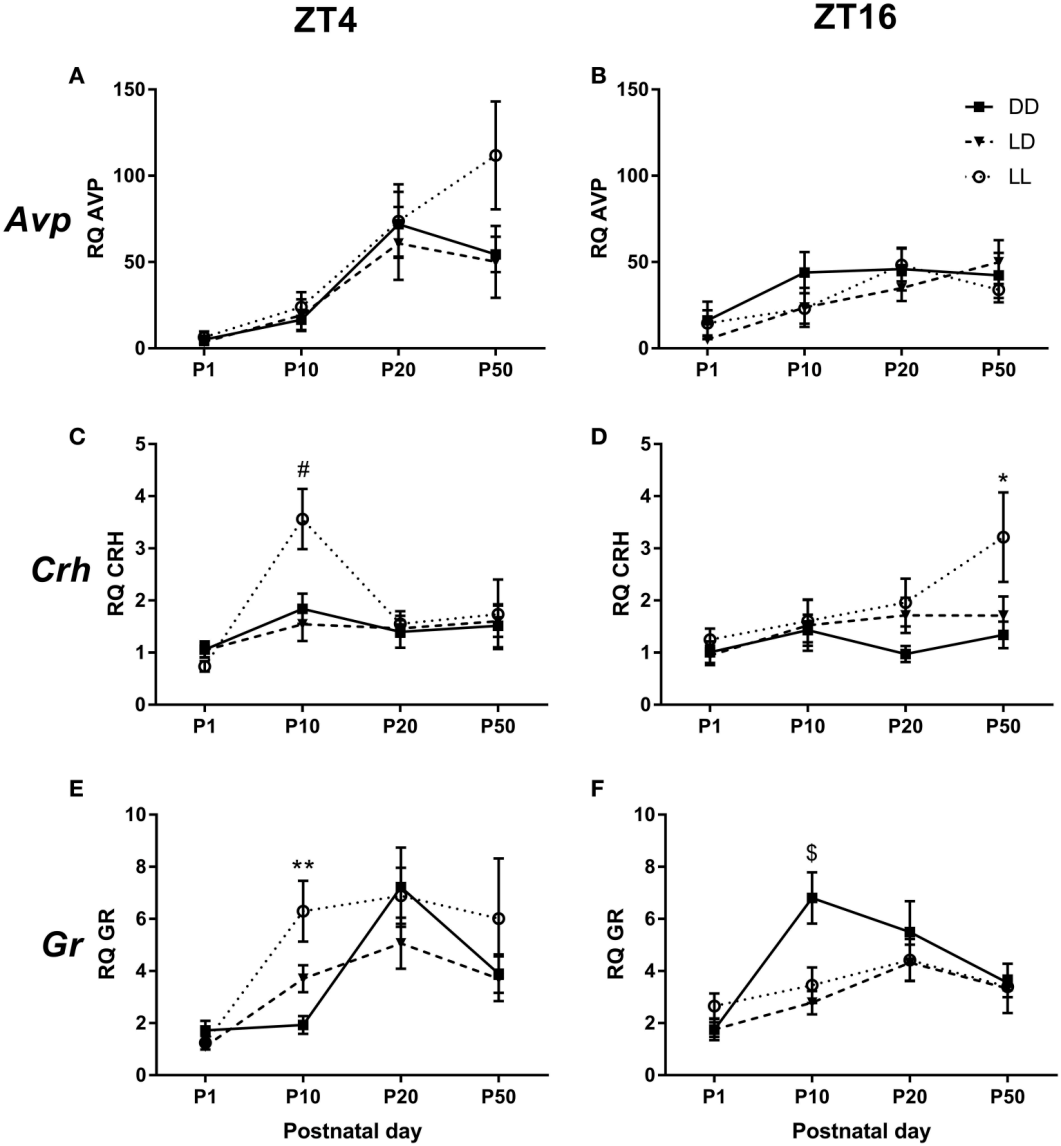
**mRNA expression in the PVN**. Relative *Arginine vasopressin* (*Avp*) **(A,B)**, *Corticotrophin-releasing hormone* (*Crh*) **(C,D)** and *Glucocorticoid receptor* (*Gr*) **(E,F)** mRNA expression in the paraventricular nucleus of the hypothalamus (PVN) of male mice raised in constant darkness (DD), 24-h light-dark cycles (LD) or constant light (LL) during the suckling stage and exposed to a LD environment thereafter. Mice were culled at Circadian/Zeitgeber Times (CT/ZT) 4 and 16, at the ages of postnatal day (P) 1, P10, P20, and P50. mRNA levels are normalized to *GapdH* expression and are relative to the P1, ZT4, LD group. ^*^p < 0.05, ^**^p < 0.005 DD vs. LL; ^#^*p* < 0.05 LL vs. LD, DD; ^$^*p* < 0.05 DD vs. LD, LL. Data are presented as average ± SEM (*n* = 4–11).

We found that *Avp* mRNA expression in the PVN was independent on the light environment to which the mice had been exposed to during the suckling stage (Figures 6A,B). There was, however, a significant effect of age [*F*_(3, 23)_ = 22.416, *p* < 0.001], with *Avp* mRNA progressively increasing as the animals aged: P1 vs. P10 (*p* < 0.05), P1 vs. P20 and P50 (*p* < 0.001), P10 vs. P20 and P50 (*p* ≤ 0.001). No significant difference was found between P20 and P50 (Figures 6A,B). There was also a significant interaction between age and ZT [*F*_(3, 23)_ = 4.433, *p* < 0.01], so that only at P20, there was a significant ZT difference, with *Avp* expression being higher at ZT4 than at ZT16 (*p* < 0.05, Figures 6A,B).

*Crh* mRNA expression was also significantly affected by age [*F*_(3, 23)_ = 7.482, *p* < 0.001], with P1 expression being lower than that at P10 and P50 (*p* < 0.001 for both comparisons, Figures 6C,D). Interestingly, a triple interaction between postnatal light environment, age and ZT was also found [*F*_(6, 23)_ = 2.371, *p* < 0.05]. Specifically, we found that at P10 and ZT4, there was an effect of postnatal light environment, with LL-raised mice showing higher *Crh* expression than LD- and DD-raised mice (*p* < 0.05 for both comparisons, Figure 6C). Moreover, we found that at P50 and ZT16, LL-raised mice had higher *Crh* mRNA expression than DD-raised mice (*p* < 0.05, Figure 6D).

Age had also a significant effect on *Gr* mRNA expression in the PVN [*F*_(3, 23)_ = 22.299, *p* < 0.001), with lower expression levels at P1 compared to P10, P20, and P50 (*p* < 0.001 for all comparisons) and higher expression levels at P20 compared to P50 (*p* < 0.005, Figures 6E,F). We also found a significant triple interaction between postnatal light environment, age and ZT [*F*_(6, 23)_ = 3.368, *p* < 0.005]: at P10 and ZT4, LL-raised mice showed higher *Gr* mRNA expression than DD-raised mice (*p* < 0.005, Figure 6E). In addition, we found that at P10 and ZT16, DD-raised mice showed higher *Gr* mRNA expression than LD-raised mice (*p* < 0.01) and LL-raised mice (*p* < 0.05, Figure 6F).

## Discussion

The aim of this study was to determine the effects of environmental light during the suckling stage on maternal behavior and stress levels, litter characteristics and growth and development of stress markers in the pup's brain. We found that, although dams exposed to LD during the suckling stage weighed more than dams exposed to DD or LL environments and dams in LL showed no significant circadian rhythm of locomotor activity, light environment did not appear to have any significant effect on stress levels or maternal behavior, including licking and grooming. Light environment did not have a major effect on litter characteristics or pup growth either. We found, however, that LL-raised pups were involved with more instances of play-fight behavior with their littermates than DD-raised pups. In addition, we found that the light environment significantly impacted *Crh* and *Gr* mRNA expression development in the PVN. Furthermore, a difference in *Crh* mRNA expression between LL- and DD-raised mice was still observed in adulthood, long after the exposure to abnormal light environments had stopped.

### Dam stress

Maternal stress can alter maternal care and subsequent stress responses of the offspring (Ivy et al., 2008). Thus, we investigated maternal HPA axis function in dams after having spent the suckling stage under abnormal light environments. Although, dams exposed to LL had very weak circadian rhythms of locomotor activity and weighed less than dams exposed to control LD cycles, their plasma corticosterone concentration, AVP and GR expression in the brain were similar, suggesting that the dams' stress systems were largely unaffected by the different light environments they experienced for 3 weeks after giving birth. This is surprising, as a change in lighting schedule has previously been reported as being accompanied with stress (LeGates et al., 2012) and LL exposure decreases corticosterone concentration in adult mice (Fonken et al., 2009; Coleman et al., 2016). On the one hand, these results could be explained by the fact that corticosterone and its receptors vary with time of day (Holmes et al., 1995; Kalsbeek et al., 2012) and we only examined one time point of the circadian cycle. On the other hand, it could be that dams have an attenuated response to stressors during the lactation period as a mechanism to protect pups. Indeed, a flattened diurnal rhythm of corticosterone has been shown in lactating dams (Windle et al., 2013). Lactating dams will, however, exhibit increased corticosterone concentrations and fragmented maternal care when exposed to strong stressful stimuli such as limited nesting material (Ivy et al., 2008), but mild stress (i.e., noise) fails to provoke a corticosterone response in dams (Windle et al., 2013). Taken together, these data suggest that LL and DD environments may be considered as mildly stressful by the dams.

### Dam behavior

Dams' circadian rhythm of locomotor activity was recorded for the first 3 weeks after the pups were born and, as expected (Brooks et al., 2014), we found that dams exposed to 24-h LD cycles were entrained to the 24 h cycles, while dams exposed to DD free-ran with a period slightly shorter than 24 h. Similarly to what we have found in previous studies (Canal-Corretger et al., 2001; Brooks et al., 2014), dams exposed to LL experienced a lengthening, together with a significant weakening of their free-running rhythm of locomotor activity. However, in this study, although the LL dams' rhythms were visible in actograms, they did not reach statistical significance. It is thus possible that dams' rhythms are generally weaker than those of adult females because of the fact that dams are recovering from giving birth, lactating and looking after their pups and therefore, their overall behavior is different to that of non-lactating adult females. This is supported by the finding that, even rhythmic dams exposed to control 24-h LD cycles or DD in this study had significantly weaker rhythms compared to those we have previously observed in adult mice (Canal-Corretger et al., 2001; Smith and Canal, 2009).

We found a significant effect of pup age on several maternal behaviors. Dam and pup behavior have been shown to change in parallel throughout the suckling stage: dams spend more time in the nest after pups are born, especially at night, but this will progressively decrease as pups grow (Yamazaki et al., 2009). We observed maternal behavior during the day (ZT/CT 4) and found that dams preferred to rest in the nest at P14, but outside the nest at P10. It is possible that the difference between the two studies is due to the fact that we only observed behavior at one time point. In our study, maternal pup retrieval behavior was higher at P6 than at P14, indicating that, as pups grew and gained autonomy, the dam did not need to bring them back to the nest for warmth and feeding. In line with this, we were able to perform the pup retrieval test at P6 and P10, but not at P14, as pups were moving freely around the cage at that age. These findings are similar to those of Yamazaki et al. (2009), who found that from P0 to P10, pups never spontaneously left the nest, but started independently venturing outside the nest and running around the cage from P11 onwards.

Maternal behavior was observed twice. The first observation started with a pup retrieval test, while the second observation did not. The initial pup retrieval test ensures that mothers are motivated to exhibit maternal behavior and offspring are motivated to suckle (Lyst et al., 2012). In agreement with this, we found that the levels of nursing and licking and grooming behaviors were augmented in the first observation compared to the second. Interestingly, the levels of licking and grooming after the pup retrieval test were lower at P14 than at P6, probably because this test worked for the P6 but not for P14 mice and therefore, the older pups needed less maternal care than the younger pups, who had been separated from their dams for a brief period of time. In line with this, we found that dams' nursing and pup retrieval behaviors were decreased at P14 compared to P6 and P10. Finally, maternal feeding and drinking were higher during the second observation period, perhaps because the dam felt hungry and thirsty after having nursed and cared for her pups after the initial pup retrieval test.

With regards to the effects of light environment on maternal behavior, our results showed that DD dams spent more time engaged in other active behavior compared to LL dams. Other active was defined as the dam moving around the cage outside the nest without engaging in any other defined behavior (e.g., feeding, resting or grooming). It is difficult to explain this difference, as all the other behaviors we measured were similar between DD, LL, and LD dams. A possible explanation for the differences we see at ZT/CT4 may be that the temporal distribution of maternal care is altered under DD conditions. If this were the case though, it would also be likely that dam behavior under LL would be altered as both DD and LL are abnormal light environments and result in changes in locomotor activity of dams. No other differences due to light environment were seen in any other maternal behaviors including licking and grooming. In rodents, maternal licking and grooming of her pups appears to be a crucial behavior that will impact the functioning of the pup HPA axis, stress reactivity and maternal behavior in adulthood (Liu et al., 1997; Pedersen et al., 2011). Therefore, our results suggest that the long-term changes in HPA axis function observed in adult mice that were raised under LL and DD environments (this study, Coleman et al., 2016) are not due to altered maternal behavior, but perhaps to a direct effect of environmental light on the stress system.

### Litter characteristics and pup growth

We found litter size and litter composition (% males) to be independent of postnatal light environment. As expected, we found that pups' body weight increased with age and a difference between males and females was observed in adulthood. In addition, we found a small effect of light environment on pup body weight: at P10, LD-raised pups weighed more than DD-raised pups, but this difference was not found later at P20 or P50. Similarly, at P20, LD-raised pups weighed less than LL-raised pups, but this difference was lost at P50. These body weight differences were minimal and observed only at 1 age and therefore, we do not believe that postnatal light environment significantly affected overall pup growth and/or development. Pups born to arrhythmic mothers have been found to have diminished growth rate (Hoshino et al., 2006). In our study, LL dams displayed weak rhythms of locomotor activity, which although were not statistically significant, were still visible in actograms for the first 2 weeks of the suckling stage. It is unclear whether the dams became arrhythmic during the last week in LL or whether their rhythms were masked by the grown pups' activity. The fact that we did not find reduced body weight/growth in LL-raised pups further supports the idea that dams exposed to LL were not totally arrhythmic. Stress has been associated with both increases and decreases in body weight (Tannenbaum et al., 2010). Moreover, maternal stress can decrease pup growth (Gao et al., 2011), and litter size and gender composition can also alter maternal care and pup growth (Musi et al., 1993; Guerra and Nunes, 2001; Chiang et al., 2002). Overall, these data suggest that neither pups nor dams were significantly stressed under LL or DD environments compared to those kept under control LD cycles. This is also supported by our findings that both maternal care and HPA axis function were overall similar between the groups.

### Pup behavior

We found no differences in the number of suck attempts between groups, supporting the finding that overall pup growth was independent on postnatal light environment. We also found that the amount of time that pups spent grooming themselves and their littermates was highest at P14. Indeed, as pups grow, the time spent resting in the nest or being nursed by their dams reduces and so they can engage in other types of behaviors (Yamazaki et al., 2009). Interestingly, we found that LL-raised pups engaged in more play-fight behavior than DD-raised pups. The reasons behind this behavior are unclear. It could be that LL-raised pups are more social so are more prone to engage in play behavior. Alternatively, it could be that LL-raised pups are more anxious and thus engage in more play-fights for comfort. However, in adult mice, we have found no differences in anxiety behavior in animals raised under LD, LL, or DD postnatal environments (Coleman et al., 2016). Furthermore, the amount of play-fight behavior in LD-raised pups was similar to that of LL- and DD-raised pups. Taken together, these results suggest that pup behavior is largely unaltered by exposure to abnormal light environments during the suckling period.

### Development of stress markers in the pup brain

#### *Avp* mRNA in the PVN

The development of the mouse HPA axis has been relatively unexplored, but studies have suggested that HPA axis responsiveness is dynamic throughout the postnatal period (Schmidt et al., 2003; Horii-Hayashi et al., 2013). We found that AVP expression progressively increased with age, with adult levels reached at P20, just before weaning occurred. Mice undergo a stress hypo-responsive (SHR) period between P1 and P12 and during this time, the HPA axis is less responsive to mild stressors to protect the developing brain from elevated glucocorticoids (Schmidt et al., 2003). Thus, it could be that down-regulation of AVP in the PVN is part of this process and therefore, after P12 when the brain is more developed and less susceptible to the damaging effects of elevated glucocorticoids, AVP levels would rise to adult levels. This is supported by our finding that *Avp* mRNA expression was higher at P20 and P50 compared to P1 and P10.

We found no effect of time in *Avp* mRNA expression in the PVN at P50, in line with a previous study in which we found no effect of time in AVP protein expression in the PVN in adult mice raised in LD, LL and DD during the suckling stage (Smith and Canal, 2009). However, an effect of time was found in *Avp* mRNA expression at P20, with day-time CT/ZT4 values being higher than those at night-time CT/ZT16. This is in contrast to a study in adult rats, which found lower *Avp* hnRNA expression in the PVN during the light period and slightly higher expression toward the end of the dark period (Watts et al., 2004). Differences in animal species, age, light conditions and/or type of RNA examined between the two studies may explain these differences. Nevertheless, since the effect of time on *Avp* mRNA expression was absent not only before at P1 and P10, but also afterwards at P50, it appears to have been transient.

We found no effect of postnatal light environment on *Avp* mRNA expression in the PVN at any of the ages examined. This is in contrast to our previous study, in which we found that AVP protein expression in the PVN at P50 was higher in DD- compared to LD- and LL-raised mice (Smith and Canal, 2009). Although, the mouse strain used and experimental design are identical between the two studies, the type of light used (fluorescent vs. LED) and its intensity (57 μW/cm^2^ vs. 330 μW/cm^2^) differed between the two studies. Alternatively, the difference could be due to the fact that we measured mRNA expression in the current study, while protein expression was measured in the Smith and Canal study (Smith and Canal, 2009). mRNA and protein correlate as little as 40%, due to the various steps between transcription and translation and half-time degradation differences (Vogel and Marcotte, 2012). Therefore, it is possible that postnatal light alters AVP protein but not mRNA expression.

#### *Crh* mRNA in the PVN

Although, we found low expression of *Crh* mRNA in the PVN at P1, this expression increased at P10 and then slightly decreased at P20, in line with Schmidt et al. (2003), who found that, during the mouse SHR period, *Crh* mRNA expression is high between P1 and P12 and then decreases between P12 and P16.

We did not find differences in *Crh* mRNA expression in the PVN due to time of day. This is in line with a previous study in rats, which found no time of day differences in *Crh* mRNA expression when the whole PVN was considered (Cai and Wise, 1996).

iAn effect of postnatal light environment on *Crh* mRNA expression was found at the ages of P10 and P50: LL-raised pups had higher expression than LD- and DD-raised mice at P10 and higher than DD-raised mice only at P50. In rodents, increased *Crh* in the PVN has been associated with depression (Wan et al., 2014). Similarly, depressed human patients exhibit elevated concentrations of CRH in blood, cerebrospinal fluid and urine (Nemeroff et al., 1984; Gold and Chrousos, 2002). These data suggest that, already at an early age, LL-raised mice may have a tendency toward depressive behavior. This is supported by our finding that adult LL-raised mice spent more time immobile in the forced swim test (Coleman et al., 2016), which is a well-established metric for despair and depressive-like behavior in rodents (Petit-Demouliere et al., 2005).

#### *Gr* mRNA in the PVN

Similarly to *Avp* and *Crh* mRNA, we found that *Gr* mRNA expression in the PVN increased between P1 and P20, but then decreased in adulthood (P50). This is in line with studies in rats, which found that GR expression was low at birth and increased slowly up until the third postnatal week (Rosenfeld et al., 1993). Similarly, *Gr* mRNA expression in the mouse hippocampus increased from birth and peaked at P12; remaining then high until P16 (Schmidt et al., 2003). The low levels of GR expression during the postnatal SHR period are thought to contribute to a reduced negative feedback, leading to reduced CRH expression and overall low HPA axis activity (Schmidt et al., 2005).

In line with previously published data (Kwak et al., 1992; Herman et al., 1993), we found no overall effect of time of day on *Gr* mRNA expression in the PVN. However, we did find a significant effect of postnatal light environment that depended on time of day, only at the age of P10: at ZT4, LL-raised mice had higher *Gr* mRNA expression than DD-raised mice, whereas at ZT16, expression was higher in DD-raised mice compared to LD- and LL-raised mice. Since in adult animals we have found no differences due to postnatal light environment in *Gr* mRNA expression (this study) or GR protein (Coleman et al., 2016) in the PVN, our finding suggests a difference in how the HPA axis develops between the groups.

## Summary and conclusions

Postnatal light environment has long-term effects on the circadian and stress systems in mice. Maternal signals such as licking and grooming and suckling have been shown to prevent long-lasting disruption of HPA responses in the offspring (Plotsky and Meaney, 1993; Caldji et al., 1998). A healthy maternal HPA axis is also critical for the correct development of the stress axis in pups (Bale, 2015). We found that dams exposed to LL or DD environments during the suckling period showed no differences in overall HPA axis function or maternal care behavior, including licking and grooming, compared to those exposed to control 24-h LD cycles. In addition, we found no differences in litter characteristics or pup growth between the groups. These results suggest that the altered stress responses observed in adult mice raised under LL or DD environments (Coleman et al., 2016) cannot be attributed to maternal behavior/stress levels, but rather, they suggest a direct effect of environmental light on the developing pup brain. This is supported by the finding, in the current study, of alterations in *Crh* and *Gr* mRNA expression in the PVN of DD- and LL-raised mice during development. It is thus possible that light experience exerts a direct effect on the offspring HPA axis. Indeed, direct projections exist between the eye and the PVN (Abrahamson and Moore, 2001). On the other hand, it could be that light experience exerts an indirect effect on the HPA axis via the circadian system, since there are strong links between the circadian and stress systems (Kalsbeek et al., 2012). Furthermore, postnatal light experience has long-lasting effects on clock gene and circadian neuropeptide expression in the SCN (Smith and Canal, 2009; Ciarleglio et al., 2011a; Brooks et al., 2014) and we have found that it can also permanently alter light-induced activation of several brain regions that receive projections from the SCN, such as the dorsomedial hypothalamic nucleus, which controls circadian regulation of corticosterone secretion (Saper et al., 2005; Brooks et al., 2011). Taken together, these data indicate that postnatal light environment can exert a direct effect on the pup's developing brain, independent of maternal influence. Further research is required to investigate the link between the effects on the developing brain and the long-lasting effects on circadian function and stress regulation that we have observed in the adult, and how these may impact the individual's future health and wellbeing.

## Author contributions

GC designed experiments, performed experiments, interpreted and analyzed data. MC designed experiments, interpreted and analyzed data, and wrote and edited the manuscript.

### Conflict of interest statement

The authors declare that the research was conducted in the absence of any commercial or financial relationships that could be construed as a potential conflict of interest.
